# An Unusual Visit from an Old Foe: Oral Presentation of Syphilis in a Teenage Patient

**DOI:** 10.1155/2021/7755914

**Published:** 2021-08-16

**Authors:** Bedirhan Tarhan, Sydur Rahman, Diana Halloran, Jeremy Sites, Avni Bhatt, Kathleen Ryan, Maritza Plaza-Verduin, Thao Vu

**Affiliations:** ^1^Department of Pediatrics, College of Medicine, University of Florida, Gainesville, FL, USA; ^2^Department of Emergency Medicine, Northwestern University Feinberg School of Medicine, Chicago, IL, USA; ^3^Department of Pediatrics, University of North Carolina School of Medicine, Chapel Hill, NC, USA; ^4^University of California, Davis School of Medicine, Sacramento, CA, USA; ^5^Department of Emergency Medicine, College of Medicine, University of Florida, Gainesville, FL, USA

## Abstract

The authors report an atypical case of secondary syphilis in an adolescent female presenting to a tertiary-care center with fever, weight loss, oral sores, painful inguinal lymphadenopathy, and transient macular rash. Given the lower prevalence of syphilis in adolescent females, this infection was not included on the initial differential diagnosis. The evolving presentation of syphilis over time complicates the diagnosis and management of these infections, as it did for the patient in this report. The authors provide a detailed discussion of the patient's clinical findings, including the protean features of syphilis infection. This case is particularly relevant to the fields of general pediatrics and pediatric hospital medicine.

## 1. Introduction

Syphilis is a genital ulcerative disease caused by the spirochete *Treponema pallidum*. It can cause serious health sequelae if not adequately treated. A 2018 CDC report showed steep and sustained increases in sexually transmitted illnesses (STIs), including syphilis, over the preceding four years [1]. Although women comprise a smaller subset of reported cases, the rate of primary and secondary syphilis among women doubled from 2013 to 2017 [1].

We report an atypical case of secondary syphilis in an adolescent female presenting with fever, weight loss, oral sores, painful inguinal lymphadenopathy, and transient macular rash. Due to the evolving presentation, diagnosis and management of syphilis are often challenging. This unusual case highlights the dynamic and, at times, enigmatic features of syphilis as well as the importance of considering the diagnosis in *all* sexually active adolescents with suggestive symptoms.

## 2. Case Report

A 15-year-old female presented to the emergency department for 4–6 weeks of mouth sores that began under her tongue but spread to her inner cheeks and lips. Two weeks prior to presentation, she experienced fevers (*T*_max_ of 38.9^o^C), sore throat, and voice changes, which subsequently resolved. She reported a nonpruritic macular rash that briefly appeared on her thighs, abdomen, and chest, though it was not present at the time of evaluation. She also had right lower quadrant abdominal pain and a mobile, discrete, and tender right inguinal lymph node. The patient experienced no change in appetite but reported a 30-pound weight loss over the preceding month. She attributed her weight loss to the pain from her mouth lesions, which limited oral intake.

Evaluation at an outside facility included a complete blood count with differential, complete metabolic panel and urinalysis, which were unremarkable aside from slightly elevated eosinophils to 8.4% (0.4–6.0% normal range). A mononuclear spot test and rapid streptococcal test, as well as blood and throat cultures, all resulted negative. After these initial studies, the patient was transferred to our institution for further evaluation and management of persistent symptoms.

On psychosocial assessment, the patient endorsed previous sexual activity with two male partners and one female partner with inconsistent use of barrier protection. On physical exam, the patient had an eruption of mucous patches and erythematous, shallow ulcers with overlying gray membranes involving the distal and lateral tongue, buccal mucosa, gingival, and inner lips. She had minimal erythema of her tonsillar pillars with no exudates or lesions. She had cheilitis sparing the vermillion border with open and healing fissures and hemorrhagic crusting. Bilateral lymphadenopathy was appreciated in the submandibular and inguinal regions. Her submandibular lymph nodes were approximately 1–1.5 cm in diameter, firm, and nontender. She was empirically treated with intravenous acyclovir due to concern for HSV gingivostomatitis and subsequently admitted to the hospital for further management.

After 48 hours of acyclovir without clinical improvement, the differential diagnosis for mucositis was broadened. Additional workup included HIV, gonorrhea, chlamydia, syphilis, and hepatitis studies. Syphilis serology resulted positive, and a reflex rapid plasma reagin (RPR) helped confirm the diagnosis of syphilis. The remainder of the workup, including HIV, gonorrhea, chlamydia, and hepatitis studies, resulted negative. Although the patient had an antinuclear antibody (ANA) titer of 1 : 320, the result was attributed to her infection with low clinical suspicion for autoimmune disorders. The patient was treated with a single intramuscular dose (2.4 million units) of benzathine penicillin G. At a ten-day follow-up appointment, the patient reported subjective improvement in symptoms. Pertinent physical exam findings included modest weight gain and complete resolution of oral lesions. Residual lymphadenopathy was noted in the right inguinal region. Repeat laboratory studies at a three-month follow-up visit revealed a negative ANA and lower RPR titer of 1 : 16.

## 3. Discussion

Syphilis is a sexually transmitted infection that can mimic different diseases with numerous and complex manifestations. For example, secondary syphilis can be mistaken for systemic lupus erythematosus (SLE) given the broad manifestations of both entities [2–4]. Autoantibody testing is performed to help diagnose patients who have clinical signs and symptoms suggestive of possible autoimmune disease. Antinuclear antibodies (ANAs) are present in many systemic autoimmune conditions such as systemic lupus erythematosus (SLE). However, as seen in our patient, a positive ANA test may also be seen with nonautoimmune or inflammatory diseases, including both acute and chronic infections. A recent study showed that various relationships exist between infections and rheumatic diseases; in particular, several patients with a positive ANA result were found to have intracellular infections including mycobacteria, syphilis, or scrub typhus [5].

Syphilis is classified into distinct stages; the primary stage is defined by a painless chancre at the site of inoculation. The secondary stage typically features a polymorphic macular or papular rash on the trunk, palms, and soles, as well as lymphadenopathy, along with constitutional symptoms of malaise, anorexia, and/or weight loss. Oral lesions are a possible, though uncommon, manifestation of secondary syphilis [6]. Secondary syphilis occurs about 6–12 weeks after the primary infection [7–9]. At that time, the spirochete enters the bloodstream and leads to generalized lymphadenopathy that is common to secondary syphilis. The spirochetes enter endothelial cells in small capillaries near the skin, leading to the appearance of the characteristic rash. The eruption may range from macular to maculopapular, follicular, or pustular, but is classically described as “raw ham” or “copper colored” [7–9]. The rash begins centrally on the trunk and spreads peripherally to the extremities, palms, soles, genitalia, and mucous membranes [8]. Our patient presented with transient, nonpruritic, macular lesions that spared the palms and soles. The rash may be associated with diffuse inflammatory involvement of the pharynx and tonsils, which may lead to a symptomatic pharyngitis. Typical lesions on the mucous membranes are described as 5–10 mm patches on the tongue, buccal mucosa, and lips; they are usually raised and painless with a central erosion/ulcer covered by a thin membrane [7–9]. Our patient presented similarly with these typical lesions as described earlier. Constitutional symptoms associated with secondary syphilis range from slight malaise to cachexia and prostration. Painless adenopathy is present in 70–85% of cases and most commonly involves the suboccipital, posterior auricular, posterior cervical, and epitrochlear lymph nodes [7]. Our patient presented with painful lymphadenopathy in her right inguinal region, which is an atypical location not commonly noted before. The tertiary stage is the most destructive and has variable clinical manifestations such as vasculitis in the vasa vasorum, tabes dorsalis from infection of the posterior columns, and gummatous lesions [6].

The presenting symptoms, stages, diagnostic testing, and appropriate antimicrobial therapy should be known to treating providers, as untreated syphilis can cause devastating cardiovascular and neurologic damage. Identification of the causative spirochete in the fluid from a chancre or condyloma lata is the most specific diagnostic technique. The spirochetes can be seen on dark-field microscopy. However, dark-field microscopy is rarely performed in clinical practice due to technical difficulties and reliable serological tests.

Syphilis infection leads to the production of nonspecific antibodies that react to cardiolipin. Binding to cardiolipin is the basis of nontreponemal tests such as RPR and VDRL. These studies, however, are not specific to syphilis. False positive reactions can occur in the setting of pregnancy, autoimmune disorders, and other infections. These tests are used to screen for syphilis, and their sensitivity is limited in early primary and late syphilis infection. RPR and VDRL can be used to monitor treatment response as they correlate fairly well with disease activity and typically decrease with treatment. Treponemal-specific tests include the EIA for antitreponemal IgG, the *T. pallidum* hemagglutination (TPHA) test, and the fluorescent treponemal antibody absorption test (FTA-abs). Treponemal-specific tests are used to confirm the diagnosis of syphilis in patients with a reactive nontreponemal test (e.g., RPR and VDRL). The treponemal tests remain positive for life, and they are not appropriate to gauge therapeutic response.

Recommended treatment is parenteral penicillin *G* for all stages of syphilis. The early stages of syphilis including primary, secondary, and early latent syphilis require a single dose of intramuscular penicillin *G* benzathine, 2.4 million units. However, late latent syphilis or tertiary syphilis requires three intramuscular doses of weekly penicillin *G* benzathine, 2.4 million units each. Alternative regimens may be used in patients who are allergic to penicillin; however, pregnant women and patients with neurosyphilis require treatment with penicillin regardless of allergy status [10].

## 4. Conclusions

Given the evolving presentation, diagnosis and management of syphilis can be challenging. Early diagnosis and rapid treatment of syphilis are essential to reducing progression to later stages and transmission to others [11]. This case demonstrates an unusual presentation of secondary syphilis in a patient with atypical age and gender demographics for the disease. The patient's extensive and painful oral lesions, as well as tender inguinal lymphadenopathy, were also very uncommon features of secondary syphilis. This report contributes to future diagnoses of atypical syphilis in adolescents, in both primary-care and the hospital setting.

## Abbreviations

STI: Sexually transmitted infection
RPR: Rapid plasma reagin
ANA: Antinuclear antibody
HSV: Herpes simplex virus
HIV: Human immunodeficiency virus.

## References

[1] Press Release 2018 STD Prevention Conference | 2018 | Newsroom | NCHHSTP | CDC 2018. https://www.cdc.gov/nchhstp/newsroom/2018/press-release-2018-std-prevention-conference.html

[2] Shatley M. J., Walker B. L., McMurray R. W. (2001). Lues and lupus: syphilis mimicking systemic lupus erythematosus (SLE). *Lupus*.

[3] Skillrud D. M., Bunch T. W. (1983). Secondary syphilis mimicking systemic lupus erythematosus. *Arthritis & Rheumatism*.

[4] Duarte J. A., Henriques C. C., Sousa C., Alves J. D. (2015). Lupus or syphilis? That is the question!. *Case Reports*.

[5] Im J. H., Chung M.-H., Park Y. K. (2020). Antinuclear antibodies in infectious diseases. *Infectious Diseases*.

[6] Rosahn P. D., Black-Schaffer B. (1943). Studies in syphilis. *Yale Journal of Biology and Medicine*.

[7] Stokes J. H. (1944). Modern clinical syphilology. *Archives of Dermatology*.

[8] Chapel T. A. (1978). The variability of syphilitic chancres. *Sexually Transmitted Diseases*.

[9] Chapel T. A. (1980). The signs and symptoms of secondary syphilis. *Sexually Transmitted Diseases*.

[10] Brown D. L., Frank J. E. (2003). Diagnosis and management of syphilis. *American Family Physician*.

[11] Cates W., Rothenberg R. B., Blount J. H. (1996). Syphilis control. *Sexually Transmitted Diseases*.

